# 900 Demographics, Outcomes, and Complications for the Homeless in Burn Unit Admissions: An Increasing Challenge?

**DOI:** 10.1093/jbcr/iraf019.431

**Published:** 2025-04-01

**Authors:** Emily Swafford, Arman Fijany, Tyler Murphy, Stephen Gondek, Anne Wagner, Robel Beyene, Elizabeth Slater

**Affiliations:** Vanderbilt Burn Center; Vanderbilt University Medical Center; Vanderbilt Burn Center; Vanderbilt University Medical Center; Vanderbilt University Medical Center; Vanderbilt University Medical Center; Vanderbilt University Medical Center

## Abstract

**Introduction:**

Burn injuries are among the most severe in terms of morbidity and mortality with socioeconomic factors often complicating management and outcomes. Individuals experiencing homelessness are especially vulnerable, facing unique challenges that impact their admission, clinical course, and outcomes. This study investigates trends in burn injuries among the homeless, focusing on demographics, outcomes, and complications.

**Methods:**

The American Burn Association Noncommercial Burn Research Dataset was queried for burn center admissions between 2012 and 2021. Individuals who identified as homeless were compared to those with confirmed housing. The primary outcome was the proportion of homeless admissions over time, and secondary outcomes included mortality, ICU admission, respiratory failure, pneumonia, DVT, and hospital length of stay (LOS). Minor complications examined were renal failure, unplanned intubation, arrhythmia, cellulitis, sepsis, alcohol withdrawal, and cardiac arrest. To reduce confounding, outcomes were adjusted for age, sex, TBSA, and inhalation injury. Odds ratios for complications were calculated using the chi-square test, and the change in the proportion of individuals experiencing homelessness relative to those with confirmed housing over time was analyzed using the Pearson correlation coefficient. Years with fewer than 100 cases were excluded. Data analysis was performed using PyCharm 3.1 software with Pandas, NumPy, and SciPy statistical modules.

**Results:**

The dataset included 159,714 admissions with 4,669 (2.9%) identifying as homeless. From 2015 to 2021, the proportion of homeless admissions rose from 1.8% to 4.3% (r = 0.98). Homeless individuals were significantly older (45.18±13.34 vs. 35.56±24.01, p < 0.01), predominantly male (p < 0.01), and more likely to be white (p < 0.01). Homelessness was linked to lower rates of unplanned intubation (p < 0.01), cellulitis (p < 0.01), sepsis (p < 0.01), renal failure (p < 0.01), and cardiac arrest (p < 0.01), but higher rates of alcohol withdrawal (p < 0.01). After adjusting for covariates, homelessness was associated with lower rates of pneumonia (OR = 0.75, p < 0.01), higher rates of ICU admission (OR = 1.33, p < 0.01), and a longer LOS (4.88 days, p < 0.01).

**Conclusions:**

This study is the largest retrospective analysis of burn admissions for the homeless population. There is a notable increase in homeless admissions compared to those with housing in recent years. Homeless burn patients exhibit lower rates of complications but higher ICU admissions and longer hospital stays.

**Applicability of Research to Practice:**

This study highlights the need for healthcare providers to adapt their approaches for homeless burn patients, acknowledging their unique challenges. By focusing on their specific outcomes, care strategies can be improved to enhance recovery for this vulnerable group.

**Funding for the Study:**

N/A

